# Large Genomic Imbalances in Brugada Syndrome

**DOI:** 10.1371/journal.pone.0163514

**Published:** 2016-09-29

**Authors:** Irene Mademont-Soler, Mel·lina Pinsach-Abuin, Helena Riuró, Jesus Mates, Alexandra Pérez-Serra, Mònica Coll, José Manuel Porres, Bernat del Olmo, Anna Iglesias, Elisabet Selga, Ferran Picó, Sara Pagans, Carles Ferrer-Costa, Geòrgia Sarquella-Brugada, Elena Arbelo, Sergi Cesar, Josep Brugada, Óscar Campuzano, Ramon Brugada

**Affiliations:** 1 Cardiovascular Genetics Center, University of Girona-IDIBGI, Girona, Spain; 2 Arrhythmia Unit, Hospital Universitario Donostia, San Sebastian, Spain; 3 Department of Medical Sciences, School of Medicine, University of Girona, Girona, Spain; 4 Gendiag SL, Barcelona, Spain; 5 Arrhythmia Unit, Hospital Sant Joan de Déu, University of Barcelona, Barcelona, Spain; 6 Arrhythmia Unit, Hospital Clinic de Barcelona, University of Barcelona, Barcelona, Spain; 7 Cardiovascular Genetics Unit, Hospital Josep Trueta, Girona, Spain; Pennsylvania State University, UNITED STATES

## Abstract

**Purpose:**

Brugada syndrome (BrS) is a form of cardiac arrhythmia which may lead to sudden cardiac death. The recommended genetic testing (direct sequencing of *SCN5A*) uncovers disease-causing SNVs and/or indels in ~20% of cases. Limited information exists about the frequency of copy number variants (CNVs) in *SCN5A* in BrS patients, and the role of CNVs in BrS-minor genes is a completely unexplored field.

**Methods:**

220 BrS patients with negative genetic results were studied to detect CNVs in *SCN5A*. 63 cases were also screened for CNVs in BrS-minor genes. Studies were performed by Multiplex ligation-dependent probe amplification or Next-Generation Sequencing (NGS).

**Results:**

The detection rate for CNVs in *SCN5A* was 0.45% (1/220). The detected imbalance consisted of a duplication from exon 15 to exon 28, and could potentially explain the BrS phenotype. No CNVs were found in BrS-minor genes.

**Conclusion:**

CNVs in current BrS-related genes are uncommon among BrS patients. However, as these rearrangements may underlie a portion of cases and they undergo unnoticed by traditional sequencing, an appealing alternative to conventional studies in these patients could be targeted NGS, including in a single experiment the study of SNVs, indels and CNVs in all the known BrS-related genes.

## Introduction

Brugada syndrome (BrS) is a form of cardiac arrhythmia, characterized by a typical electrocardiographic pattern of ST segment elevation in leads V1 to V3, and incomplete or complete right bundle branch block [1]. A common presentation of BrS is syncope, which is caused by fast polymorphic ventricular tachycardia. Such syncope typically occurs in the third and fourth decade of life, and usually at rest or during sleep. In some cases, tachycardia does not terminate spontaneously, and it may degenerate into ventricular fibrillation and lead to sudden death [2].

BrS exhibits an autosomal dominant pattern of inheritance, with incomplete penetrance and variable expressivity. Currently, its global prevalence is estimated at 3–5 in 10 000 people, although the incidence is higher in Southeast Asian countries than in the United States and Europe. The syndrome is genetically heterogeneous and can arise from pathogenic variants in at least 19 different genes [3]. The major gene associated with BrS is *SCN5A*, which encodes for the α-subunit of the voltage-gated cardiac sodium channel Na_V_1.5. Screening for pathogenic variants in this gene uncovers mutations in approximately 20% of BrS patients [4,5]. An additional 15% of patients can be molecularly diagnosed if the minor genes described as causing BrS are included in the genetic analysis (*ABCC9*, *CACNA1C*, *CACNA2D1*, *CACNB2*, *FGF12*, *GPD1L*, *HCN4*, *KCND3*, *KCNE1L*, *KCNE3*, *KCNH2*, *KCNJ8*, *PKP2*, *RANGRF*, *SCN1B*, *SCN2B*, *SCN3B*, *SCN10A*, *SLMAP* and *TRPM4*) [3,6,7]. Hence, a causal genetic variant is not found in a high percentage of patients with BrS.

Genetic testing of BrS patients generally involves sequencing of protein-coding portions and flanking intronic regions of *SCN5A*, according to current ESC Guidelines [8]. The diagnosis is usually performed by direct sequencing, which does not enable the detection of large genomic imbalances (Copy Number Variants, CNVs), which could explain a portion of BrS cases. Although for other arrhythmogenic disorders (such as Long QT syndrome) a relevant contribution of CNVs to the disease has been described (2–11.5%) [9], limited information is available about their contribution to BrS. In 2011, Eastaugh *et al*. [10] reported the first BrS patient (with a concomitant conduction system disease) with a large rearrangement, consisting of a deletion of exons 9 and 10 of *SCN5A*. This rearrangement underwent unnoticed by traditional sequencing and was detected by a quantitative approach. In addition, it was predicted to cause no functional protein to appear in the membrane, resulting in haploinsufficiency. Such finding led the authors to suggest that assessment of CNVs in *SCN5A* should be considered as a standard part of genetic testing in BrS patients. However, after this first report of a CNV in BrS, only three series have been published regarding the frequency of CNVs in *SCN5A* in genotype-negative BrS patients, and no further large deletions or duplications were identified [11–13]. Although the cohorts studied were relatively small (N = 38; N = 68; and N = 37), the authors concluded that such imbalances do not seem to have a major contribution to BrS. On the other hand, the role of CNVs in minor genes related to BrS is a completely unexplored field.

In this report, we present the largest screening for CNVs in *SCN5A* in genotype-negative BrS patients. We also assess, for the first time, the contribution of large genomic imbalances in BrS-associated minor genes.

## Materials and Methods

### Study population

Two hundred and twenty non-related patients of European descent with a definite BrS phenotype and negative genetic results (for Single Nucleotide Variants -SNVs- and small insertions/deletions -indels-) were studied to detect CNVs in *SCN5A*. For a portion of cases (N = 63), minor genes related to BrS were also screened. The mean age of the patients at the time of clinical diagnosis was 43.53±13.54 years, and at the time of genetic ascertainment 48.69±14.52 years. The 79% of patients were males. The genetic analyses previously performed in the 220 BrS patients that led to their classification as genotype-negative were: a) in 120 cases, conventional Sanger sequencing of *SCN5A*; b) in 37 cases, Sanger sequencing of the following BrS-related genes: *CACNA1C*, *CACNB2*, *GPD1L*, *HCN4*, *KCNE1L*, *KCNE3*, *KCND3*, *KCNJ8*, *RANGRF*, *SCN1B*, *SCN2B*, *SCN3B* and *SCN5A* (results published by Selga et al., 2015 [13]); c) in 43 cases, Next-Generation Sequencing (NGS) analysis using a custom panel that included the genes *CACNA1C*, *CACNB2*, *GPD1L*, *HCN4*, *PKP2* and *SCN5A* (NGS panel 1); and d) in 20 cases, NGS analysis with a custom panel that included the genes *ABCC9*, *CACNA1C*, *CACNA2D1*, *CACNB2*, *GPD1L*, *HCN4*, *KCND3*, *KCNE1L*, *KCNE3*, *KCNJ8*, *PKP2*, *RANGRF*, *SCN1B*, *SCN2B*, *SCN5A*, *SLMAP* and *TRPM4* (NGS panel 2). All assays were performed on total genomic DNA isolated from blood or saliva samples using Chemagen MSM I (PerkinElmer, Germany). For the genes mentioned, only coding regions and flanking intronic sequences were analyzed. NGS panels were developed by Gendiag.exe SL and commercialized by Ferrer InCode as SudD inCode^®^.For patients in the categories a) and b) (N = 157), CNVs were only assessed in *SCN5A* by Multiplex ligation-dependent probe amplification (MLPA). For patients in the categories c) and d) (N = 63), CNVs in *SCN5A* as well as the minor genes included in each custom panel were studied after analysis of NGS data with an algorithm developed in our laboratory to detect large genomic imbalances. In cases where a CNV was detected, clinical data and blood samples from relatives were analyzed for segregation studies and interpretation of results. Clinical investigation of relatives included medical history, clinical examination and 12-lead electrocardiogram (ECG). The study was approved by the ethical committee of Hospital Universitari Dr. Josep Trueta de Girona (Spain) and conformed to the ethical guidelines of the Declaration of Helsinki 2008. Informed written consent was obtained from all patients.

### Detection of CNVs by MLPA

MLPA analysis was carried out in 157 BrS patients using the commercially available SALSA MLPA P108 *SCN5A* probemix (MRC-Holland, Amsterdam, The Netherlands). This kit contains probes for each exon of *SCN5A* and one probe upstream of this gene (isoform NM_198056.2). Remarkably, for exon 1 the probe is within the intron (beginning 209 nt after exon 1), and for exon 28 two probes are included. For each experiment, 3 reference DNAs from healthy individuals were used. The MLPA protocol was carried out according to manufacturer’s instructions (MRC-Holland). After the multiplex PCR reaction, electrophoresis was performed using the ABI3130XL genetic analyzer with LIZ500 size standard (both from Applied Biosystems, Waltham, MA, USA), and results were analyzed using Coffalyser.Net (MRC-Holland). A reduction or increase in the relative signal strength of >30% was considered as a deletion or duplication of the locus, respectively. For confirmation, each CNV identified was studied by an alternative method.

### Detection of CNVs by NGS

Sequencing data from 63 BrS patients that were prepared with NGS custom panels 1 and 2 (including *SCN5A* and several BrS-associated minor genes) were analyzed for detection of CNVs. Sample libraries had been prepared following the SureSelect XT Target Enrichment System for Illumina Paired-End Sequencing Library protocol (Agilent Technologies, Santa Clara, CA, USA). Indexed libraries had been sequenced in a ten-sample pool on a MiSeq platform (Illumina, San Diego, CA, USA), with 2x75 bp reads length. For the detection of CNVs, a bioinformatic algorithm developed in our laboratory was used. In brief, the approach focuses on capturing significant differences between the expected and the obtained normalized coverages for a given sample in every exon of the genes of interest. Raw coverage is first normalized by the amount of DNA yielded for each sample in the run. Then the insert size and the low probe affinity bias for targeted regions with a too high GC content (>75%) or too low GC content (<45%) are corrected. Finally, the ratio between each sample and a built-in baseline is evaluated. If the ratio falls outside a signal-to-noise window and is greater or lower than the duplication or deletion cut-offs (0.45 and -0.8, respectively), the gain or loss is inferred. For confirmation, each CNV identified was studied by an alternative method.

## Results

### CNVs in *SCN5A*

**Fig 1:**
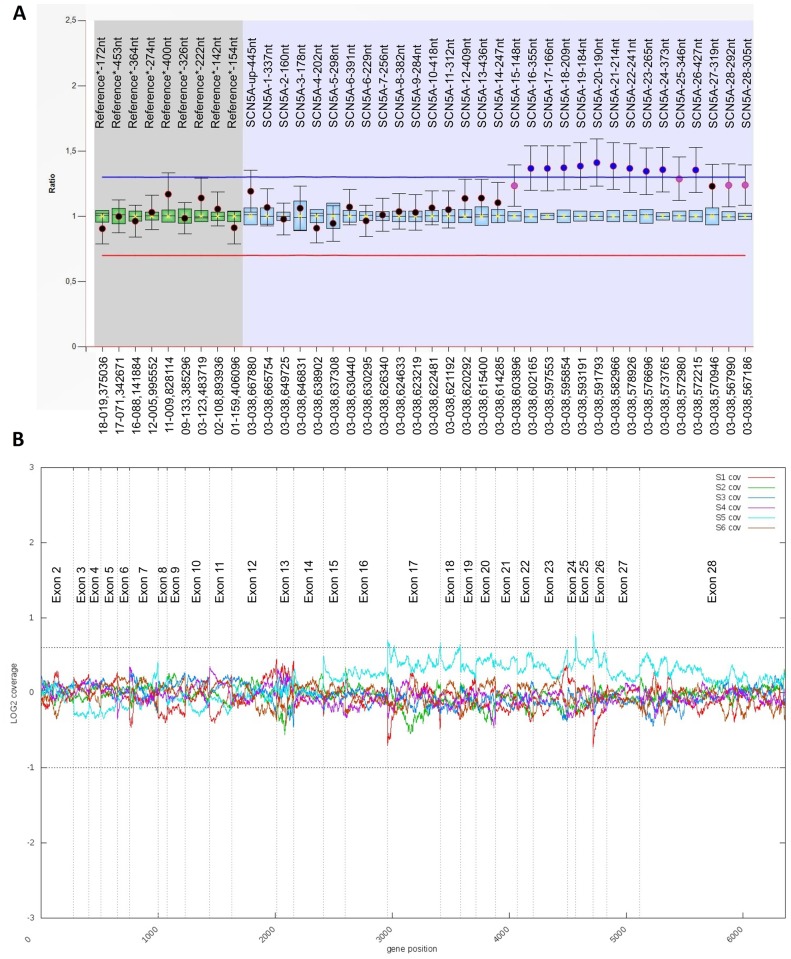
*SCN5A* duplication in a Brugada syndrome patient. Results of Multiplex ligation-dependent probe amplification (**A**) and Next-Generation Sequencing (**B**, patient in light blue) showing the duplication from exon 15 to 28 of *SCN5A*. Exon numbering according to isoform NM_198056.

Among the 220 genotype-negative BrS patients investigated for CNVs in *SCN5A* by NGS or MLPA, one large genomic imbalance was detected. Thus, the detection rate for CNVs in *SCN5A* in this cohort was 0.45%. The imbalance consisted of a large duplication spanning from exon 15 to exon 28 of *SCN5A*. The rearrangement was first detected by MLPA (repeated four times) and then confirmed by NGS (using the custom NGS panel 1) (Fig 1A and 1B). For both techniques, DNA obtained from the same fresh whole blood sample was used. The NGS analysis did not reveal any SNV, indel or CNV different from that of *SCN5A* that could potentially explain the BrS phenotype. Interestingly, the imbalance was found in a mosaic state, as the signals of both techniques for all duplicated exons were lower than expected for a heterozygous duplication (ratio of 1.5 for MLPA and log2 ratio of 0.6 for NGS). Moreover, the signals for the first and last exons of the duplicated region were lower than those for the other exons, suggesting that the rearrangement could be more complex than a typical duplication. To further characterize this rearrangement, new fresh whole blood and saliva samples were requested 11 years latter to obtain DNA and RNA. When MLPA (performed in blood and saliva) and NGS (performed in blood, with custom panel 2) were performed in these new samples, the imbalance was not detected. Again, no SNVs, indels and CNVs that could potentially explain the BrS phenotype were detected. To discard sample swapping, the NGS results for SNVs and indels of the first and second blood samples were compared. The analysis of the genes included in both panels (as each sample was prepared with a different NGS panel) revealed the identical 107 SNVs and 3 indels in both samples, so the possibility of sample swapping was discarded. For further studying the case, a dermal biopsy was collected from the patient and kept frozen until DNA extraction (using Chemagen MSM I, PerkinElmer). The rearrangement was not detected in this tissue.

**Fig 2:**
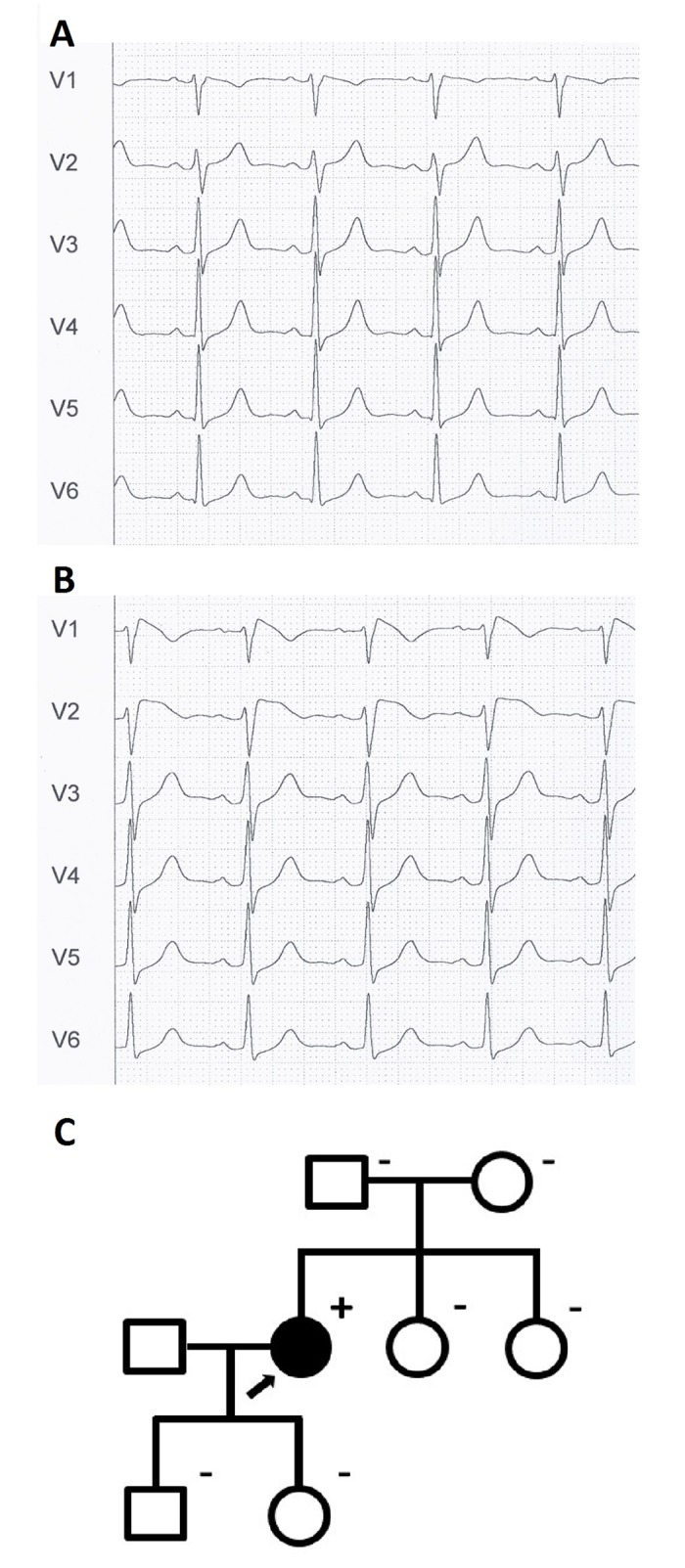
Clinical data from the patient with the Copy Number Variant in *SCN5A*. **A.** Basal electrocardiogram. **B.** Electrocardiogram after flecainide test, showing type I Brugada pattern. **C.** Family pedigree. The proband is indicated by an arrow. Subjects affected and unaffected by BrS are indicated by solid and open symbols, respectively. Genetic status for the *SCN5A* rearrangement is indicated by a superindex (+ or -).

The patient with the *SCN5A* rearrangement was a 48-year-old woman diagnosed with BrS after a syncopal episode. On ECG examination, a type I Brugada pattern was detected in V1 and V2. Electrophysiological study showed that she was inducible into non-sustained ventricular arrhythmias. Flecainide test was positive (Fig 2A and 2B, basal ECG and flecainide test, respectively). She was implanted with a defibrillator. Clinical evaluation and genetic study by MLPA of the index case relatives (parents, two siblings, one son and one daughter) revealed that they presented neither a BrS phenotype nor the *SCN5A* rearrangement (Fig 2C).

### CNVs in minor genes

No large exon duplications or deletions in the BrS-associated minor genes screened were found in any of the 63 genotype-negative BrS patients studied by NGS. Specifically, in 43 patients the analysis involved the genes *CACNA1C*, *CACNB2*, *GPD1L*, *HCN4*, and *PKP2*; and in 20 patients the genes *ABCC9*, *CACNA1C*, *CACNA2D1*, *CACNB2*, *GPD1L*, *HCN4*, *KCND3*, *KCNE1L*, *KCNE3*, *KCNJ8*, *PKP2*, *RANGRF*, *SCN1B*, *SCN2B*, *SLMAP*, and *TRPM4*. NGS raw data have been uploaded to Figshare (https://dx.doi.org/10.6084/m9.figshare.3564141.v3 and https://dx.doi.org/10.6084/m9.figshare.3565980.v1).

## Discussion

Large-scale genomic imbalances are a significant contributor to the molecular pathology of a number of different genetic disorders [9,14]. To determine the involvement of such rearrangements to BrS, we performed the largest screening for CNVs in *SCN5A* in genotype-negative BrS patients and assessed, for the first time, their prevalence in BrS-associated minor genes. We also evaluated the clinical value of investigating these large rearrangements in BrS patients as part of the routine molecular testing.

Only one large genomic imbalance was detected after screening *SCN5A* for CNVs in our series of 220 genotype-negative BrS patients (for SNVs and indels in *SCN5A* and in some of them also in BrS-associated minor genes) (0.45%). These results indicate that although such rearrangements in *SCN5A* are not very common among BrS patients, they are found at least in some of the cases who test negative for disease-causing variants in BrS-related genes using conventional sequencing methods. The obtained results are in agreement with published data. A previous study reported a BrS patient (with a concomitant conduction system disease) with a large deletion in *SCN5A* that was considered the underlying cause of the phenotype [10]. However, the other three published series evaluating CNVs in *SCN5A* in BrS patients revealed a frequency of such rearrangements of 0% (cohort sizes N = 38, N = 68 and N = 37)[11–13]. The latter study was published by our group and the cases are included in the present series.

The CNV identified in *SCN5A* in a BrS patient consisted of a large rearrangement involving a duplication of exons 15 to 28 in a mosaic state. According to the signals obtained from MLPA and NGS analysis, the imbalance is probably more complex than a typical tandem duplication. Unfortunately, the rearrangement could not be further characterized since it was not detected in the blood, saliva and skin samples received 11 years latter. However, we suggest that the imbalance may be present in heart, being responsible for the observed BrS phenotype as: a) the CNV involves *SCN5A* gene, which is the most significant gene so far described as causing BrS; b) the rearrangement is *de novo* and not transmitted to the offspring, and the index case is the only family member affected by BrS; c) radical variants (including nonsense variants, indels, frameshift variants, and variants affecting splice sites) are a well-known cause of BrS, as they lead to loss of sodium channel function. The rearrangement identified could also result in a complete loss of function of the affected allele, leading to the BrS phenotype. The option of the duplication being inserted in another region of the genome (thus not altering *SCN5A* structure and expression) can not be discarded. However, only 2.5% of clinically relevant duplications are found to be insertional translocations [15].

In relation to the mosaic state of the rearrangement, to our knowledge there are no published BrS patients with pathogenic genetic variants (SNVs, indels or CNVs) found in mosaic state. However, mosaicism for CNVs has been nowadays largely reported in both healthy individuals and in association with disease [16–18]. A particularity of our case is that the mosaicism in blood disappeared within a period of eleven years. Cases with genetic analysis being repeated in the same individual after a period of several years are rare, but there are some publications reporting the follow-up of mosaic cases which have revealed differences in the proportions of mosaicism)[19–24]. Furthermore, some of these studies report cases with complete disappearance of the normal or the abnormal cell line, a phenomenon first described by La Marche et al. in 1967 as “disappearing mosaicism” [25]. Regarding this issue, two previous reports deserve special attention. In 1984, Motegi and Minoda [20] reported three patients with retinoblastoma and 13q14 deletion mosaicism for which a significant decrease in the proportion of the abnormal cell line was observed in peripheral lymphocytes over time. On the other hand, Morales et al. reported in 2007 [23] a newborn with a partial duplication of chromosome 7q and the complementary deletion, in whom the cell line with the deletion completely disappeared in blood after the first year of life. It is important to highlight that although mosaicism may disappear from blood, it could remain in other more stable tissues (such as skin, brain or heart). Blood cells are unfortunately an unstable source of genetic material given multiple rounds of self-renewal during hematopoiesis and, moreover, the diversity of the clonal lineages that give rise to circulating blood cells appears to decrease with age [18]. Considering all these data, in our case we hypothesize that, although disappearing from blood, the abnormal cell line with the duplication of several exons of *SCN5A* may be present in the heart, resulting in the observed BrS phenotype. Our hypothesis is consistent with previous studies, such as those performed in right ventricular outflow tract tachycardia and atrial fibrillation, which showed that certain genetic variants found in cardiac tissue could be completely absent in blood [26–28]. The fact that the *SCN5A* duplication was not detected in skin may be explained by the embryological origin of the tissues under consideration. Whereas blood and cardiac tissue are derived exclusively from the mesoderm, skin is formed from both mesoderm and ectoderm. All these data suggest that a portion of sporadic BrS cases may arise from pathogenic variants found in the heart and not detectable in other tissues. Molecular diagnosis of these cases is important, as if the mosaicism involves the germline there is a high risk for recurrence of the disease. Thus, we believe that further research in this field will prove beneficial for better understanding of the role of mosaicism in BrS, and for determining the appropriate tissue for diagnosis of sporadic BrS cases.

On the other hand, in the present study we explored, for the first time, the presence of CNVs in BrS-minor genes as responsible of the phenotype in 63 genotype-negative BrS patients. Specifically, the minor genes investigated were *ABCC9*, *CACNA1C*, *CACNA2D1*, *CACNB2*, *GPD1L*, *HCN4*, *KCND3*, *KCNE1L*, *KCNE3*, *KCNJ8*, *PKP2*, *RANGRF*, *SCN1B*, *SCN2B*, *SLMAP*, and *TRPM4*. No large imbalances were detected, suggesting that such imbalances in these genes do not probably have a major contribution to BrS. However, further studies with larger cohorts are needed to elucidate the precise involvement of CNVs in these genes in BrS patients.

In most laboratories, the current approach for molecular diagnosis of BrS patients involves exclusively conventional Sanger sequencing of *SCN5A*, which does not enable the screening for CNVs. Although SNVs and indels in *SCN5A* are the main recognized cause of BrS, 20 other genes have been associated with the disease so far, and CNVs may also explain a portion of cases [3–10]. As the identification of the genetic variant causing the BrS phenotype is the only way to offer an accurate genetic counseling to families and to identify at-risk family members (with the ultimate aim of preventing sudden death), we believe that the best approach for a comprehensive study of BrS patients would be targeted NGS using a panel including all known BrS-related genes, and investigating the presence of CNVs as part of the genetic analysis. Under this scenario, all well-known genetic causes of BrS could be explored. However, as the contribution of the known minor genes and CNVs to BrS seems to be low, a significant proportion of individuals with a clinical diagnosis of BrS will still remain without a positive genetic diagnosis. Further efforts need to be done to describe other causes of BrS, which may include genetic variants in non-coding regions (i.e. promoters and other regulatory regions, introns, and untranslated regions), novel pathogenic alterations in as yet unknown genes and, as previously discussed, cardiac-specific mosaicism.

In conclusion, our results after performing the largest screening for CNVs in *SCN5A* and minor genes in BrS patients reveal that such rearrangements are not a common finding among genotype-negative BrS patients. However, as these rearrangements may underlie a portion of cases and they undergo unnoticed by traditional sequencing, we believe that an appealing alternative to conventional studies in BrS patients would be targeted NGS, including in a single experiment the study of SNVs, indels and CNVs in all the known BrS-related genes.

## References

[1] Brugada P, Brugada J Right bundle branch block, persistent ST segment elevation and sudden cardiac death: a distinct clinical and electrocardiographic syndrome. A multicenter report. J Am Coll Cardiol. 1992; 20: 1391–1396. 1309182 10.1016/0735-1097(92)90253-j

[2] Napolitano C, Priori SG. Brugada syndrome. Orphanet J Rare Dis. 2006; 1: 35 16972995 10.1186/1750-1172-1-35 PMC1592481

[3] Sarquella-Brugada G, Campuzano O, Arbelo E, Brugada J, Brugada R. Brugada syndrome: clinical and genetic findings. Genet Med. 2016; 18: 3–12. 10.1038/gim.2015.35 25905440

[4] Priori SG, Napolitano C, Gasparini M, Pappone C, Della Bella P, Giordano U, et al Natural history of Brugada syndrome: insights for risk stratification and management. Circulation. 2002; 105: 1342–1347. 11901046 10.1161/hc1102.105288

[5] Kapplinger JD, Tester DJ, Alders M, Benito B, Berthet M, Brugada J, et al An international compendium of mutations in the SCN5A-encoded cardiac sodium channel in patients referred for Brugada syndrome genetic testing. Heart Rhythm. 2010; 7: 33–46. 10.1016/j.hrthm.2009.09.069 20129283 PMC2822446

[6] Hennessey JA, Marcou CA, Wang C, Wei EQ, Tester DJ, Torchio M, et al FGF12 is a candidate Brugada syndrome locus. Heart Rhythm. 2013; 10: 1886–1894. 10.1016/j.hrthm.2013.09.064 24096171 PMC3870051

[7] Wang Q, Ohno S, Ding WG, Fukuyama M, Miyamoto A, Itoh H, et al Gain-of-function KCNH2 mutations in patients with Brugada syndrome. J Cardiovasc Electrophysiol. 2014; 25: 522–530. 10.1111/jce.12361 24400717

[8] Priori SG, Blomstrom-Lundqvist C, Mazzanti A, Blom N, Borggrefe M, Camm J, et al 2015 ESC Guidelines for the management of patients with ventricular arrhythmias and the prevention of sudden cardiac death: The Task Force for the Management of Patients with Ventricular Arrhythmias and the Prevention of Sudden Cardiac Death of the European Society of Cardiology (ESC). Endorsed by: Association for European Paediatric and Congenital Cardiology (AEPC). Eur Heart J. 2015; 36: 2793–2867. 10.1093/eurheartj/ehv316 26320108

[9] Campuzano O, Sarquella-Brugada G, Mademont-Soler I, Allegue C, Cesar S, Ferrer-Costa C, et al Identification of Genetic Alterations, as Causative Genetic Defects in Long QT Syndrome, Using Next Generation Sequencing Technology. PLoS One. 2014; 9: e114894 10.1371/journal.pone.0114894 25494010 PMC4262446

[10] Eastaugh LJ, James PA, Phelan DG, Davis AM. Brugada syndrome caused by a large deletion in SCN5A only detected by multiplex ligation-dependent probe amplification. J Cardiovasc Electrophysiol. 2011; 22: 1073–1076. 10.1111/j.1540-8167.2010.02003.x 21288276

[11] Koopmann TT, Beekman L, Alders M, Meregalli PG, Mannens MM, Moorman AF, et al Exclusion of multiple candidate genes and large genomic rearrangements in SCN5A in a Dutch Brugada syndrome cohort. Heart Rhythm. 2007; 4: 752–755. 17556197 10.1016/j.hrthm.2007.02.021

[12] Garcia-Molina E, Lacunza J, Ruiz-Espejo F, Sabater M, Garcia-Alberola A, Gimeno JR, et al A study of the SCN5A gene in a cohort of 76 patients with Brugada syndrome. Clin Genet. 2013; 83: 530–538. 10.1111/cge.12017 22984773

[13] Selga E, Campuzano O, Pinsach-Abuin ML, Perez-Serra A, Mademont-Soler I, Riuro H, et al Comprehensive Genetic Characterization of a Spanish Brugada Syndrome Cohort. PLoS One. 2015; 10: e0132888 10.1371/journal.pone.0132888 26173111 PMC4501715

[14] Miller DT, Adam MP, Aradhya S, Biesecker LG, Brothman AR, Carter NP, et al Consensus statement: chromosomal microarray is a first-tier clinical diagnostic test for individuals with developmental disabilities or congenital anomalies. Am J Hum Genet. 2010; 86: 749–764. 10.1016/j.ajhg.2010.04.006 20466091 PMC2869000

[15] Newman S, Hermetz KE, Weckselblatt B, Rudd MK. Next-generation sequencing of duplication CNVs reveals that most are tandem and some create fusion genes at breakpoints. Am J Hum Genet. 2015; 96: 208–220. 10.1016/j.ajhg.2014.12.017 25640679 PMC4320257

[16] Piotrowski A, Bruder CE, Andersson R, Diaz de Stahl T, Menzel U, Sandgren J, et al Somatic mosaicism for copy number variation in differentiated human tissues. Hum Mutat. 2008; 29: 1118–1124. 10.1002/humu.20815 18570184

[17] O'Huallachain M, Karczewski KJ, Weissman SM, Urban AE, Snyder MP. Extensive genetic variation in somatic human tissues. Proc Natl Acad Sci U S A. 2012; 109: 18018–18023. 10.1073/pnas.1213736109 23043118 PMC3497787

[18] Campbell IM, Shaw CA, Stankiewicz P, Lupski JR. Somatic mosaicism: implications for disease and transmission genetics. Trends Genet. 2015; 31: 382–392. 10.1016/j.tig.2015.03.013 25910407 PMC4490042

[19] Holmgren G, Jagell S, Lagerkvist B, Nordenson I. A pair of siblings with diastrophic dysplasia and E trisomy mosaicism. Hum Hered. 1984; 34: 266–268. 6479995 10.1159/000153477

[20] Motegi T, Minoda K. A decreasing tendency for cytogenetic abnormality in peripheral lymphocytes of retinoblastoma patients with 13q14 deletion mosaicism. Hum Genet. 1984; 66: 186–189. 6714979 10.1007/BF00286598

[21] McCorquodale MM, Bowdle FC. Two pregnancies and the loss of the 46,XX cell line in a 45,X/46,XX Turner mosaic patient. Fertil Steril. 1985; 43: 229–233. 3967782 10.1016/s0015-0282(16)48378-0

[22] Priest JH, Rust JM, Fernhoff PM. Tissue specificity and stability of mosaicism in Pallister-Killian +i(12p) syndrome: relevance for prenatal diagnosis. Am J Med Genet. 1992; 42: 820–824. 10.1002/ajmg.1320420615 1554021

[23] Morales C, Madrigal I, Esque T, de la Fuente JE, Rodriguez JM, Margarit E, et al Duplication/deletion mosaicism of the 7q(21.1 —> 31.3) region. Am J Med Genet A. 2007; 143A: 179–183. 10.1002/ajmg.a.31570 17163539

[24] Gravholt CH, Friedrich U, Nielsen J. Chromosomal mosaicism: a follow-up study of 39 unselected children found at birth. Hum Genet. 1991; 88: 49–52. 1959925 10.1007/BF00204928

[25] La Marche PH, Heisler AB, Kronemer NS. Disappearing mosaicism. Suggested mechanism is growth advantage of normal over abnormal cell population. R I Med J. 1967; 50: 184–189. 5231789

[26] Lerman BB, Dong B, Stein KM, Markowitz SM, Linden J, Catanzaro DF. Right ventricular outflow tract tachycardia due to a somatic cell mutation in G protein subunitalphai2. J Clin Invest. 1998; 101: 2862–2868. 10.1172/JCI1582 9637720 PMC508877

[27] Gollob MH, Jones DL, Krahn AD, Danis L, Gong XQ, Shao Q, et al Somatic mutations in the connexin 40 gene (GJA5) in atrial fibrillation. N Engl J Med. 2006; 354: 2677–2688. 10.1056/NEJMoa052800 16790700

[28] Thibodeau IL, Xu J, Li Q, Liu G, Lam K, Veinot JP, et al Paradigm of genetic mosaicism and lone atrial fibrillation: physiological characterization of a connexin 43-deletion mutant identified from atrial tissue. Circulation. 2010; 122: 236–244. 10.1161/CIRCULATIONAHA.110.961227 20606116

